# Successful ageing among a national community-dwelling sample of older adults in India in 2017–2018

**DOI:** 10.1038/s41598-021-00739-z

**Published:** 2021-11-12

**Authors:** Supa Pengpid, Karl Peltzer

**Affiliations:** 1 ASEAN Institute for Health Development, Mahidol University, Salaya, Phutthamonthon, Nakhon Pathom Thailand; 2 Department of Research Administration and Development, University of Limpopo, Turfloop, South Africa; 3 Department of Psychology, University of the Free State, Bloemfontein, South Africa; 4 Department of Psychology, College of Medical and Health Science, Asia University, Taichung, Taiwan

**Keywords:** Diseases, Health care

## Abstract

This study aimed to determine the prevalence and correlates of successful ageing in older community-dwelling adults in India. The cross-sectional sample included 21,343 individuals (≥ 65 years) from the Longitudinal Ageing Study in India (LASI) Wave 1 in 2017–2018. Successful ageing was assessed utilizing a multidimensional concept, including five components: (1) absence of major illness, (2) free of disability, (3) no major depressive disorder, (4) social engagement and (5) life satisfaction. Overall, 27.2% had successful ageing, including 83.3% had no major diseases, 51.0% free from disability, 91.8% had no major depressive disorder, 73.6% were socially engaged and 74.6% had high life satisfaction. In the adjusted logistic regression analysis, male sex (Adjusted Odds Ratio-AOR 1.40, 95% Confidence Interval-CI 1.21–1.26), married (AOR 1.48, 95% CI 1.22–1.79), having formal education (AOR 1.47, 95% CI 1.23–1.74), high subjective socioeconomic status (AOR 1.61, 95% CI 1.29–2.01), urban residence (AOR 1.42, 95% CI 1.19–1.70), Sikhs (AOR 1.76, 95% CI 1.38–2.24), high physical activity (AOR 1.65, 95% CI 1.38–1.97), and daily Yoga practice (AOR 1.34, 95% CI 1.11–1.61) increased the odds of successful ageing, while increasing age (AOR 0.96, 95% CI 0.94–0.79), poor childhood health (AOR: 0.47, 95% CI 0.29–0.75), and underweight (AOR 0.70, 95% CI 0.61–0.81) decreased the odds of successful ageing. Almost one in three older adults in India were successfully ageing. Factors associated with successful ageing included, male sex, married, having formal education, high subjective socioeconomic status, urban residence, Sikhs, physical activity, Yoga practice, younger age, good childhood health, and not having underweight.

## Introduction

“India is projected to become the world's most populous nation by 2028, with a population of some 1.45 billion”^1^.

With longevity and declining fertility rates, the population of older persons (60 years and above) is globally growing faster than the general population. The share of population over the age of 60 is projected to increase from 8 percent in 2015 to 19 percent in 2050. By the end of the century, the elderly will constitute nearly 34 percent of the total population in the country^2^.

There has been a shift in focus from “how long” to “how healthy” or “how successful” older adults live^3^. The “National Policy on Older Persons” (NPOP) in India has been instituted for improving quality of life of elderly in India^2^. One means to be used for evaluating quality of life of older adults in India is by assessing and monitoring successful ageing (SA). Definitions of SA or healthy ageing include “survival to a specific age, being free of chronic diseases, autonomy in activities of daily living, well-being, good quality of life, high social participation, only mild cognitive or functional impairment, and little or no disability”^4^. Common domains assessed in SA include “physical capability, cognitive function, metabolic and physiological health, psychological well-being, and social well-being,” which significantly predict morbidity and mortality^5^. Commonly used models of successful ageing include the biomedical model of successful ageing (BMSA) and a multidimensional concept of successful ageing (MMSA)^6,7^. The BMSA may include five components: “no major disease, high cognitive functioning, high physical functioning, no disability and active engagement with life”^6,7^, and MMSA may include five components, such as no major disease, no disability, mental well-being, social engagement, and life satisfaction^8^.

Using the MMSA, the prevalence of SA was among older adults (≥ 65 years) 18.6% in China^8^, in South Korea (≥ 65 years) 25.2%^8^, and in 15 European countries (≥ 50 years) 23.5%^9^. Using the BMSA, the prevalence of SA was among older adults (≥ 60 years) in China 13.2%^10^, in Singapore (≥ 60 years) 25.4%^7^, in three East Asian countries (China, Korea, and Japan) (65 and 75 years) 17.6%^11^. The proportion of five components of MMSA was, for example in China (≥ 65 years) “no major illness 75.1%, no disability 86.0%, no depression symptom 75.2%, active social/productive engagement 51.2%, and life satisfaction 57.1%”^8^, and the prevalence of five components of BMSA was, for example in China (≥ 60 years) no major diseases 41.7%, no disability 92.1%, high cognitive functioning 54.2%, high physical functioning 70.2%, and active engagement with life 46.0%^10^. To our knowledge, we could not find any study on SA among older adults in India, which prompted this study. Such data could give us a better understanding on successful ageing at a national level and make cross-cultural comparisons.

Sociodemographic factors associated with SA may include younger age^8,11,12^, male sex^8,11,12^, married^12^, spouse accompany^8^, higher education^3,7,8,10,12–15^, higher income/wealth^8,12–14^, higher childhood wealth^15^, ethnicity^7^, region^10^, and urban residence^16^. In addition, various health behaviours have been found associated with SA, including not smoking^17^, alcohol drinking^8^, physical activity^12,17–19^, healthy diet^20^, cognitive activity^12^, and a normal body mass index^17^. Studies on SA among older adults have largely been conducted in high-income countries, except for China. Considering differences in socioeconomic contexts, culture, retirement and leisure in low resourced countries, such as in India, an understanding of SA among older adults in India is important. Therefore, this study aimed to determine SA in older community-dwelling adults in India in 2017–2018.

## Method

### Sample and procedures

This secondary data analysis utilized data from the cross-sectional and nationally representative “Longitudinal Ageing Study in India (LASI) Wave 1, 2017–2018”; “the overall household response rate is 96%, and the overall individual response rate is 87%”^21^. In a household survey, “interview, physical measurement and biomarker data were collected from individuals aged 45 and above and their spouses, regardless of age”^21^. We restricted our sample to those 65 years and older in this analysis. The study was approved by the “Indian Council of Medical Research (ICMR) Ethics Committee and written informed consent was obtained from the participants”^21^. All methods were carried out in accordance with relevant guidelines and regulations.

### Measures

#### Successful ageing

SA was assessed utilizing a MMSA, including five components: (1) absence of major illness, (2) free of disability, (3) no major depressive disorder, (4) social engagement and (5) life satisfaction^8^.

*Absence of major illnesses* were sourced from the questions, “Has any health professional ever told you that you have…?”: (1) “chronic lung disease such as asthma, chronic obstructive pulmonary disease/chronic bronchitis or other chronic lung problems; (2) cancer or malignant tumor; (3) chronic heart diseases such as coronary heart disease (heart attack or myocardial infarction), congestive heart failure, or other chronic heart problems; and (4) stroke”^21^.

*Free of disability* was measured based on “Activities of Daily Living (ADL) (6 items) and Instrumental Activities of Daily Living (IADL) (7 items)”^22,23^, (Cronbach alpha 0.89) and defined as 0 ADL and 0 or 1 IADL difficulty.

*Major depressive disorder* was assessed with the Composite International Diagnostic Interview short form (CIDI-SF)^24^. Study respondents were required to “endorse either anhedonia or depressed mood for most of the day for most of a 2-week period or more,” and those who fulfilled this criterion “completed an additional seven symptoms: lost interest, feeling tired, change in weight, trouble with sleep, trouble concentrating, feeling down, and thoughts of death”^25^. “Those with a score ≥ 3 was considered to meet the criteria for having MDD in the previous 12 months; MDD symptomology scores ranged from 0 to 7”^25^.

Social engagement was measured with 6 items, e.g., “Eat-out-of-house (restaurant/hotel)”^21^. Responses were coded 1 = daily to at least once a month and 0 = rarely/once a year or never (Cronbach’s alpha 0.71). Social engagement was defined as any positive response to any of the 6 items.

*Life satisfaction* was sourced from the 5-item Satisfaction With Life Scale (SWLS)^26^. Higher scores (20–35) (range 5–35) indicated higher life satisfaction. (Cronbach’s alpha of the SWLS was 0.89 in this sample).

SA was further assessed utilizing BMSA, including five components: (1) absence of major illness (chronic lung disease, heart disease, stroke, cancer, diabetes and major depressive disorder), (2) free of disability (0 difficulty with ADL), (3) high cognitive functioning, (4) high physical function, and (5) social engagement. *High cognitive functioning* was defined as an above median total score on tests involving immediate and delayed word recall, serial 7 s, and backward counting (0–27)^9,15^. *High physical function* was defined as 0–1 difficulty with the following five activities: “(1) waking 100 yards; (2) climbing one flight of stairs without resting; (3) stooping, kneeling or crouching; (4) pulling or pushing large objects; (5) lifting or carrying weights over 5 kilos like a heavy bag of groceries”^21^.

#### Covariates and confounders

*Sociodemographic variables* consisted of level of education (none, ≥ 1 years), age in years, sex (male, female), residential status, religion, and marital status (married, and not married, including never married, live-in relationship, widowed, divorced, separated, and deserted). Subjective socioeconomic status was assessed with the question, “Please imagine a ten-step ladder, where at the bottom are the people who are the worst off—who have the least money, least education, and the worst jobs or no jobs, and at the top of the ladder are the people who are the best off—those who have the most money, most education, and best jobs. Please indicate the number (1–10) on the rung on the ladder where you would place yourself”^21^. Steps 1 to 3 on the socioeconomic ladder were defined as low, 4–5 as medium, and 6–10 as high socioeconomic status.

*Poor childhood health* was assessed with the question, “Would you say your childhood health was very good, good, fair, poor or very poor on the basis of what you remember, or what you heard or perceived from your parents?”) (Coded poor or very poor = 1)^21^.

*Childhood poverty* was sourced from the item, “Now think about your family when you were growing up, from birth to age 16. Compared to other families in your community, would you say your family during that time was pretty well off financially, about average, or poor?”^21^, and defined as “poor” (vs. average or pretty well off financially).

*Current tobacco use* was assessed from (1) “Do you currently smoke any tobacco products (cigarettes, bidis, cigars, hookah, cheroot, etc.)?” and/or (2) “Do you use smokeless tobacco (such as chewing tobacco, gutka, pan masala, etc.)?” (Yes, No)^21^.

*Heavy alcohol use* was assessed with the question, “In the last 3 months, how frequently on average, have you had at least 5 or more drinks on one occasion?”^21^ and defined as “one to three days per month, one to four days per week, five or more days per week, or daily.”

*Physical activity* (PA) was assessed with the questions (1) “How often do you take part in sports or vigorous activities, such as …: everyday, more than once a week, once a week, one to three times a month, or hardly ever or never?” (2) “On the days you did vigorous activity, how much time did you usually spend doing any vigorous activity? (___minutes)”, (3) “How often do you take part in sports or activities that are moderately energetic such as…?” and (4) “How much time did you usually spend doing any moderate activity on an average in a day?”^21^. The participants were classified into 4 levels of PA according to their waking duration throughout the week: a) no PA (0 min/week), b) low- PA (1 to < 150 min/week moderate intensity or “1–74 min/week vigorous intensity or 1–149 min/week moderate + vigorous intensity; whereby time in vigorous activity is doubled”), c) moderate PA (150–300 min/week moderate intensity or 75–149 min/week vigorous intensity or “150–300 min/week moderate + vigorous intensity; whereby time in vigorous activity is doubled”), and high PA (> 300 min/week moderate PA or “ > 150 min/week vigorous intensity or > 300 min/week moderate + vigorous intensity; whereby time in vigorous activity is doubled”)^27,28^.

*Frequency of Yoga practice* was assessed with the question*, “*How often do you engage in any of the following activities like yoga, meditation, asana, pranayama or similar?” Responses were trichotomized into “1 = hardly ever or never, 2 = One to three times a month, once a week, or more than once a week, and 3 = every day”^21^.

*Anthropometry* “Height and weight of adults were measured using the Seca 803 digital scale”^21^. “Body Mass Index = BMI was calculated according to Asian criteria: underweight (< 18.5 kg/m^2^), normal weight (18.5–22.9 kg/m^2^), overweight (23.0–24.9 kg/m^2^), class I obesity (25.0–29.9 kg/m^2^), and class II obesity (≥ 30.0 kg/m^2^)”^29^.

### Data analysis

Descriptive statistics were applied to describe sociodemographic information, health indicators and SA. Pearson Correlation was used to calculate correlations between SA components. Unadjusted and adjusted logistic regression was utilized to assess associations between sociodemographic, health behaviour and MMSA and BMSA. *P* < 0.05 was accepted as significant, missing values were excluded, and no multi-collinearity was found. Statistical analyses were conducted using “STATA software version 15.0 (Stata Corporation, College Station, TX, USA)”, taking the complex study design into account.

## Results

### Sample characteristics

The sample included 21,343 older adults (65 years and older, median 70 years), 52.2% were female and 47.8% male. Majority (69.9%) of the participants were rural dwellers, 58.4% had no schooling, 55.2% were married, 82.0% were Hindus, and 39.6% had low subjective socioeconomic status. Few of participants (1.7%) had poor childhood health, and 42.2% had childhood poverty. One third of the older adults (33.0%) were currently using tobacco, 2.3% in heavy alcohol use, 38.1% in no physical activity, 9.2% engaged in daily Yoga practice, and 28.4% were underweight. Overall, 27.2% had successful ageing, including 83.3% had no major diseases, 51.0% free from disability, 91.8% had no major depressive disorder, 73.6% were socially engaged and 74.6% had high life satisfaction (see Table 1).

**Table 1:** Sample and successful ageing characteristics in percent among older adults (≥ 65 years) in India, 2017–2018.

Variable	Sample	Successful ageing components					Successful ageing
		No major diseases	No disability	No depressive disorder	Social engagement	Life satisfaction	
**Sociodemographic factors**							
All	21,343	83.3	51.0	91.8	73.6	74.6	27.2
Age in years: Median (interquartile range)	70 (9)	70 (8)	70 (8)	70 (8)	70 (8)	70 (8)	69 (7)
Sex							
Female	10,877 (52.2)	85.1	42.4	90.8	70.9	73.0	21.9
Male	10,466 (47.8)	81.2	60.6	92.9	76.6	76.4	33.1
Marital status							
Not married	9115 (44.8)	84.4	41.8	90.5	68.7	70.7	19.9
Married	12,222 (55.2)	82.4	58.6	92.8	77.6	77.8	33.1
Education							
No schooling	11,919 (58.4)	85.6	44.5	90.9	64.8	69.9	20.2
≥ 1 year	9424 (41.6)	79.9	60.3	93.1	86.1	81.1	37.0
Socioeconomic status							
Low	7203 (39.6)	85.5	49.1	88.7	66.7	63.4	20.1
Medium	7876 (36.1)	82.8	53.5	93.3	77.1	79.8	29.9
High	5295 (24.3)	82.2	56.2	94.6	84.1	85.1	35.1
Residential status							
Rural	14,032 (69.9)	84.6	48.9	90.9	67.4	73.0	23.9
Urban	7311 (30.1)	80.1	56.1	94.0	88.3	78.4	35.2
Religion							
Hindu	15,520 (82.0)	83.9	50.7	91.8	73.6	74.5	27.0
Muslim	2534 (11.4)	79.4	49.2	92.0	69.4	75.6	25.5
Christian	2203 (2.8)	80.4	55.5	93.0	79.8	70.3	29.9
Sikh	684 (2.0)	84.6	68.4	91.5	80.0	81.9	39.9
Other	401 (1.8)	80.4	51.1	90.5	81.4	73.7	30.6
**Childhood factors**							
Poor childhood health	326 (1.7)	79.1	33.7	73.1	72.0	70.2	12.9
Childhood poverty	8268 (42.2)	84.1	48.6	89.6	67.5	71.1	24.1
**Health behaviour**							
Current tobacco use	6525 (33.0)	85.8	53.8	91.2	71.8	72.6	27.1
Heavy alcohol use	618 (2.3)	87.6	59.6	93.4	70.4	71.5	27.7
Physical activity							
No	8269 (38.1)	78.9	42.2	90.4	65.1	75.0	21.0
Low	3162 (14.2)	83.7	51.9	92.4	79.7	73.1	26.5
Moderate	1798 (8.4)	86.9	53.6	94.7	77.2	78.8	30.9
High	7927 (39.2)	86.7	58.7	92.2	79.0	73.9	32.4
Yoga							
Never	18,042 (87.1)	83.4	49.1	91.9	71.4	73.5	25.3
< daily	786 (3.8)	84.3	64.7	90.3	87.2	79.8	40.9
Daily	2319 (9.2)	81.9	63.8	91.2	88.8	82.8	40.0
Body mass index							
Normal	7249 (38.8)	85.3	56.0	92.7	75.7	74.6	30.0
Underweight	4738 (28.4)	85.2	47.7	90.0	64.3	69.2	19.7
Overweight/obesity	6668 (32.8)	80.7	53.2	93.3	85.0	80.3	31.6

### Successful ageing by biomedical and multidimensional model

Table 2 provide an overview of the prevalence of each SA component stratified by SA models and by age groups. In both models the prevalence of SA declined with age. Looking at the different SA components, no disease and life satisfaction did not decline with age, while all other SA components declined with age (no disability, high cognitive functioning, high physical function, social engagement and no major depressive disorder). Using the MMSA, 83.3% had no disease, 51.0% no disability, 91.8% no major depressive disorder, 73.6% social engagement, and 74.6% life satisfaction, and using the BMSA, 71.0% had no disease, 72.9 no disability, 55.4% high cognitive functioning, 31.3% high physical functioning, and 73.6% social engagement (see Table 2).

**Table 2:** Successful ageing by biomedical and multidimensional model.

Successful ageing model and components	Age group			Biomedical model	Multidimensional model
	65–74	75–84	85 +	65 +	65 +
**Biomedical model**					
No disease: chronic lung disease, heart disease, stroke, cancer, diabetes, and major depressive disorder	71.5	69.4	71.4	71.0	
No disability (0 ADL)	78.3	64.1	51.8	72.9	
High cognitive functioning	59.1	49.6	33.6	55.4	
High physical functioning	35.7	24.5	13.7	31.3	
Social engagement	77.8	68.0	52.0	73.6	
Total	13.0	7.3	2.6	11.0	
**Multidimensional model**					
No disease: chronic lung disease, heart disease, stroke, and cancer	84.0	80.9	84.2		83.3
No disability (0 ADL & 0–1 IADL)	57.3	40.8	26.6		51.0
No major depressive disorder	92.4	91.4	87.6		91.8
Social engagement	77.8	68.0	52.0		73.6
Life satisfaction	75.0	73.5	73.9		74.6
Total	30.9	21.0	11.2		27.2

### Correlations between successful ageing and its components

Table 3 show zero-order correlations between multidimensional SA, biomedical SA and their five components. The highest correlations were between no disability, social engagement and life satisfaction with multi-dimensional SA, and between high physical functioning and high cognitive function with biomedical SA (see Table 3).

**Table 3:** Correlations between successful ageing components.

Multidimensional SA and its components	Successful ageing	No major illness	No disability	No depressive disorder	Social engagement	Life satisfaction
Successful ageing	1.00					
No major illness	0.27	1.00				
No disability	0.58	0.11	1.00			
No depressive disorder	0.18	0.05	0.13	1.00		
Social engagement	0.36	− 0.01	0.12	0.07	1.00	
Life satisfaction	0.36	0.02	0.06	0.11	0.08	1.00

Biomedical SA and its components	Successful ageing	No major illness	No disability	High cognitive functioning	High physical functioning	Social engagement
Successful ageing	1.00					
No major illness	0.22	1.00				
No disability	0.20	0.08	1.00			
High cognitive functioning	0.31	− 0.05	0.12	1.00		
High physical functioning	0.50	0.10	0.32	0.10	1.00	
Social engagement	0.19	− 0.03	0.13	0.20	0.06	1.00

### Successful aging by state

Using the MMSA the highest prevalence of SA was in Mizoram (58.9%), followed by Nagaland (52.7%), Gujarat (44.0%), and Puducherry (45.3%), and the lowest was in Karnataka (18.7%), followed by Telangana (20.9%), and West Bengal (19.7%), while using the BMSA the highest prevalence of SA was in Puducherry (27.8%), Mizoram (25.3%), and Chandigarh (23.4%), and the lowest in Odisha (6.9%), West Bengal (7.1%), and Arunachal Pradesh (7.9%) (see Table 4).

**Table 4:** Successful ageing by multidimensional model (MMSA) and biomedical model (BMSA) by state (N = 21,343).

State in India	MMSA	BMSA
	%	%
Jammu & Kashmir	20.4	9.8
Himachal Pradesh	35.5	19.9
Punjab	38.6	18.4
Chandigarh	41.9	23.4
Uttarakhand	34.7	14.3
Haryana	33.6	15.5
Dehli	33.6	15.4
Rajasthan	26.4	14.3
Uttar Pradesh	26.8	8.8
Bihar	26.1	12.0
Arunachal Pradesh	41.2	7.9
Nagaland	52.7	14.8
Manipur	40.0	24.4
Mizoram	58.9	25.3
Tripura	34.5	8.8
Meghalaya	42.6	16.0
Assam	31.0	11.4
West Bengal	20.7	7.1
Jharkhand	21.5	7.6
Odisha	30.6	6.9
Chhattisgarh	34.6	12.0
Madhya Pradesh	24.7	12.3
Gujarat	44.0	13.7
Daman & Diu	40.6	11.5
Dadra & Nagar Haveli	38.9	12.9
Maharashtra	29.9	9.1
Andhra Pradesh	24.5	15.1
Karnataka	18.7	10.6
Goa	38.5	10.7
Lakshadweep	38.3	17.8
Kerala	30.4	8.6
Tamil Nadu	30.2	14.6
Puducherry	45.3	27.8
Andaman & Nicobar Islands	36.8	8.5
Telangana	20.9	11.2

### Associations with successful ageing

In the adjusted logistic regression analysis, male sex (Adjusted Odds Ratio-AOR 1.40, 95% Confidence Interval-CI 1.21–1.26), married (AOR 1.48, 95% CI 1.22–1.79), having formal education (AOR 1.47, 95% CI 1.23–1.74), high subjective socioeconomic status (AOR 1.61, 95% CI 1.29–2.01), urban residence (AOR 1.42, 95% CI 1.19–1.70), Sikhs (AOR 1.76, 95% CI 1.38–2.24), high physical activity (AOR 1.65, 95% CI 1.38–1.97), and daily Yoga practice (AOR 1.34, 95% CI 1.11–1.61) were positively associated with MMSA, while increasing age (AOR 0.96, 95% CI 0.94–0.79), poor childhood health (AOR 0.47, 95% CI 0.29–0.75), and underweight (AOR 0.70, 95% CI 0.61–0.81) were negatively associated with associated with MMSA. The correlates for BMSA were for the most part (8 variables) like MMSA. In addition, childhood poverty and overweight/obesity were negatively associated with BMSA and urban residence was not associated with BMSA (see Table 5).

**Table 5:** Associations with successful ageing.

Variable	Multi-dimensional concept		Biomedical concept	
	Crude odds ratio (95% CI)	Adjusted odds ratio (95% CI)	Crude odds ratio (95% CI)	Adjusted odds ratio (95% CI)
**Sociodemographic factors**				
Age in years	0.94 (0.93, 0.95)***	0.96 (0.94, 0.97)***	0.93 (0.91, 0.94)***	0.93 (0.91, 0.95)***
Sex				
Female	1 (Reference)	1 (Reference)	1 (Reference)	1 (Reference)
Male	1.77 (1.58, 1.98)***	1.40 (1.21, 1.61)***	2.41 (2.06, 2.82)***	2.17 (1.73, 2.72)***
Marital status				
Not married	1 (Reference)	1 (Reference)	1 (Reference)	1 (Reference)
Married	1.99 (1.74, 2.28)***	1.48 (1.22, 1.79)***	2.04 (1.71, 2.42)***	1.22 (0.99, 1.49)
Education				
No schooling	1 (Reference)	1 (Reference)	1 (Reference)	1 (Reference)
≥ 1 year	2.32 (1.99, 2.71)***	1.47 (1.23, 1.74)***	2.55 (2.11, 3.09)***	1.55 (1.24, 1.94)***
Socioeconomic status				
Low	1 (Reference)	1 (Reference)	1 (Reference)	1 (Reference)
Medium	1.69 (1.39, 2.06)***	1.40 (1.14, 1.73)***	1.53 (1.21, 1.94)***	1.25 (0.99, 1.60)
High	2.15 (1.73, 2.67)***	1.61 (1.29, 2.01)***	2.04 (1.60, 2.62)***	1.55 (1.22, 1.97)***
Residential status				
Rural	1 (Reference)	1 (Reference)	1 (Reference)	1 (Reference)
Urban	1.73 (1.44, 2.07)***	1.42 (1.19, 1.70)***	1.41 (1.14, 1.75)**	1.19 (0.92, 1.54)
Religion				
Hindu	1 (Reference)	1 (Reference)	1 (Reference)	1 (Reference)
Muslim	0.93 (0.75, 1.15)	0.93 (0.71, 1.21)	0.87 (0.68, 1.11)	0.80 (0.61, 1.06)
Christian	1.16 (0.93, 1.44)	1.23 (0.96, 1.56)	1.43 (0.92, 2.22)	1.50 (0.95, 2.36)
Sikh	1.80 (1.45, 2.24)***	1.76 (1.38, 2.24)***	1.68 (1.26, 2.24)***	1.52 (1.07, 2.16)*
Other	1.20 (0.76, 1.89)	1.26 (0.76, 2.10)	0.70 (0.40, 1.22)	0.78 (0.45, 1.38)
**Childhood factors**				
Poor childhood health	0.39 (0.24, 0.64)***	0.47 (0.29, 0.75)**	0.56 (0.29, 1.07)	–
Childhood poverty	0.93 (0.81, 1.07)	–	0.65 (0.52, 0.81)***	0.73 (0.59, 0.92)**
**Health behaviour**				
Current tobacco use	0.99 (0.88, 1.12)	–	0.98 (0.83, 1.17)	–
Heavy alcohol use	1.02 (0.75, 1.39)	–	1.14 (0.75, 1.73)	–
Physical activity				
No	1 (Reference)	1 (Reference)	1 (Reference)	1 (Reference)
Low	1.36 (1.11, 1.65)**	1.15 (0.87, 1.52)	1.62 (1.28, 2.04)***	1.39 (1.06, 1.82)*
Moderate	1.68 (1.32, 2.14)***	1.47 (1.13, 1.91)**	1.87 (1.42, 2.45)***	1.59 (1.20, 2.11)***
High	1.80 (1.55, 2.09)***	1.65 (1.38, 1.97)***	1.85 (1.53, 2.25)***	1.70 (1.37, 2.12)***
Yoga				
Never	1 (Reference)	1 (Reference)	1 (Reference)	1 (Reference)
< daily	2.04 (1.50, 2.78)***	1.44 (1.03, 2.00)*	1.33(0.98, 1.82)	0.95 (0.68, 1.35)
Daily	1.97 (1.70, 2.29)***	1.34 (1.11, 1.61)**	2.03 (1.69, 2.45)***	1.38 (1.11, 1.71)**
Body mass index				
Normal	1 (Reference)	1 (Reference)	1 (Reference)	1 (Reference)
Underweight	0.57 (0.50, 0.65)***	0.70 (0.61, 0.81)***	0.66 (0.54, 0.80)***	0.83 (0.67, 1.02)
Overweight/obesity	1.08 (0.91, 1.28)	0.87 (0.74, 1.02)	0.81 (0.65, 0.98)*	0.73 (0.60, 0.89)***

*CI* Confidence Interval.

***p < 0.001;**p < 0.01; *p < 0.05.

## Discussion

To our knowledge, this study is the first to assess the prevalence and factors associated with SA among older adults (≥ 65 years) in a national community-based sample in India in 2017–2018. Using the MMSA, we found that almost one in three older adults (27.2%) in India were successfully ageing, which is higher than in China (18.6%, ≥ 65 years) in China^8^ and like a study in South Korea (25.2%, ≥ 65 years)^8^, and in 15 European countries (≥ 50 years) 23.5%^9^. Possible reasons for the higher MMSA in India than in China may be related to the use of different indicators, e.g., in China depressive symptoms were measured that have a higher prevalence than in India measuring major depressive disorder, and lower awareness of chronic diseases in India than in China. Using the BMSA, we found that more than one in ten (11.0%) older adults in India were successfully ageing, which is similar to China (13.2%, ≥ 60 years)^10^, and lower than in Singapore (19.6%, ≥ 65 years)^7^, and in three East Asian countries (China, Korea, and Japan) (17.6%, 65 and 75 years)^11^. Comparing the assessment of SA with MMSA and BMSA, this study found in line with previous research^8,9^ that the rates of MMSA were higher than BMSA. The more flexible MMSA may be more useful for targeting identified deficiencies in public health interventions^9^.

Using the MMSA, 83.3% had no disease, 51.0% no disability, 91.8% no major depressive disorder, 73.6% social engagement, and 74.6% life satisfaction in this study, which compares to the China (≥ 65 years) study, as follows, no major illness 75.1%, no disability 86.0%, no depression symptom 75.2%, active social/productive engagement 51.2%, and life satisfaction 57.1%^8^. No disability was in this study lower than in the China (≥ 65 years) study, and social engagement and life satisfaction was higher in this study than in the China (≥ 65 years) study^8^. Using the BMSA, we found in this study that 71.0% had no disease, 72.9 no disability, 73.6% social engagement, 31.3% high physical functioning, and 55.4% high cognitive functioning, which compares with the China (≥ 60 years) study of active engagement with life 46.0%, high physical function 70.2%, high cognitive functioning 54.2%, no disability 92.1%, and no major diseases 41.7%^10^. The proportion of older adults with no disease and social engagement were higher in this study than in the China (≥ 60 years) study^10^, while high physical function and no disability were higher in the China (≥ 60 years) study^10^ than in this study. Analysing the different components of SA by age groups, we found that the decline with age was stronger for social engagement, high cognitive functioning, high physical function, and no disability, while this was less pronounced for no disease, no major depressive disorder and life satisfaction. Similar findings were identified in an investigation among older adults in Germany^12^.

We found that male sex, married, having formal education, high subjective socioeconomic status, no childhood poverty, urban residence, Sikhs, physical activity, Yoga practice, younger age, good childhood health, not having underweight and overweight/obesity were associated with MMSA and/or BMSA. Consistent with previous research^8,11,12^, male sex was found to be associated with SA, which may be related to gender paradox in health (women living with worse health longer than men)^8^. These gender differences seem to be mainly attributed to men having higher no disability and social engagement than women. The found gender differences are consistent with research showing lower functional health among older women than men in India^30^. In addition, it may be possible that women experience greater barriers to access health care services than men in India^31^. In addition, younger age and being married was associated with SA in this study, which concurs with previous findings^8,11,12^. The negative association between age and SA is expected due to the biological, functional and cognitive decline with ageing^7^.

In line with various studies^3,7,8,10,12–15^, higher socioeconomic status (in childhood and adulthood), higher education and urban residence were associated with SA in this study. Higher education may increase health behaviour, health care seeking, and cognitive functioning, and thus increase SA^7^. Likewise, higher economic status shows better access to economic resources, which may help in enabling to engage in better health and dietary behaviour^14,32^. Urban residence may be associated with higher educational and economic status and better access to health care, all of which could increase SA^16^. No disability and social engagement were lower among older adults residing rural compared to urban areas in this study. Social participation should be promoted among older adults in rural areas in India. Furthermore, we found ethnic and regional differences in the prevalence of SA, as found in previous research^7,10^. Compared to Hindus, Sikhs were likely to have a higher prevalence of SA in this study. Sikhs had the highest rates of no major diseases, no disability and life satisfaction, which contributed to their overall SA. The highest prevalence of SA was in the Indian states of Mizoram, Nagaland, Gujarat, Puducherry and Chandigarh, and the lowest was in Karnataka, Telangana, West Bengal, Arunachal Pradesh, and Odisha. Some of these state differences may be attributed to differences in the level of economic development and health outcomes. For example, life expectancy ranged from 56 years in Madhya Pradesh to 74 years in Kerala^33^, and in this vain, states with a higher life expectancy, such as Delhi (74.7 years), and Punjab (72.4 years) had also higher rates of SA, Delhi 33.6% MMSA and 15.4% BMSA, and Punjab 38.6% MMSA and 18.4% BMSA, while states with a lower life expectancy, such as Madhya Pradesh (66.0 years) and Karnataka (69.2 years) had also lower rates of SA, Madhya Pradesh 24.7% MMSA and 12.3% BMSA, and Karnataka 18.7% MMSA and 10.6% BMSA^34^.

Consistent with studies^12,17–19^, this study showed a positive association between physical activity, daily Yoga practice and SA. In a systematic review Yoga practice was found to be associated with better subjective health and health behaviours^35^. Research has provided evidence that physical activity improves health^19,36^, prevents several chronic conditions^19^, is beneficial to mental health^37,38^, and increases life satisfaction^39^, cognitive functioning^40,41^, and functional ability^19,40^, all of which may contribute to better SA. While some previous research^8,17^ found an association between not smoking, alcohol drinking and SA, we did not find a significant association. Furthermore, a body mass index not in the normal range (underweight and overweight/obesity decreased the odds of SA, which is in agreement with another research^17^. Overweight/obesity has been shown to lead to chronic physical conditions^42^, and underweight was associated with poorer health outcomes^41^, and low BMI was associated with non-survival^42^. The association between physical activity, Yoga practice and body weight status with SA may be utilized in targeting modifiable risk factors to promote SA^7^. Similar to a study among older adults in Germany^12^, this study did not find any association between non-tobacco use and SA. It is possible that non-tobacco use is more specifically beneficial for physical health, rather than SA^12^. The “National Programme for the Health Care of the Elderly (NPHCE)” in India addresses: “many health concerns of elderly (such as increasing incidence of non-communicable disease) which demand the strengthening and reorientation of the primary health care system to the special needs of the elderly, improving geriatric care at all levels, and promoting the concept of healthy ageing”^2^.

Study limitations include the cross-sectional design, the assessment of some variables by self-report. A bias may be less for diagnosed chronic conditions than for self-reported health. Some variables, such as dietary behaviour and mental or cognitive activity^12,20^, that have been shown to influence SA were not assessed and should be included in future research. The statistical models were adjusted for various confounding variables, but findings may still have been confounded by other variables, such as psychological coping resources, not included in the analyses. Furthermore, the study focused on community-dwelling older adults and excluded institutionalised persons.

## Conclusion

Almost one in three older adults in India were successfully ageing. Factors associated with successful ageing included, male sex, married, having formal education, high subjective socioeconomic status, urban residence, Sikhs, physical activity, Yoga practice, younger age, good childhood health, and not having underweight. Since LASI was designed as a longitudinal study, future research may want to evaluate and monitor the predictive value of MMSA and BMSA in the older adult population in India.
